# Infantile suprasellar tumor diagnosed as a pineoblastoma RB1 subgroup and treatment challenges: A pediatric SNO Molecular Tumor Board

**DOI:** 10.1093/noajnl/vdac092

**Published:** 2022-06-14

**Authors:** Jeffrey A Rubens, Craig Erker, Holly Lindsay, Ben Ho, Bryan Li, Eric Bouffet, Alan Cohen, Charles Eberhart, Birgit Ertl-Wagner, Anita Mahajan, Stergios Zacharoulis, Annie Huang, Roger Packer

**Affiliations:** Division of Pediatric Oncology, Sidney Kimmel Comprehensive Cancer Center, School of Medicine, Johns Hopkins University, Baltimore, Maryland, USA; Division of Hematology/Oncology, Department of Paediatrics, IWK Health Centre and Dalhousie University, Halifax, Nova Scotia, Canada; Division of Pediatric Hematology and Oncology, Department of Pediatrics, Texas Children’s Hospital/Baylor College of Medicine, Houston, Texas, USA; Division of Hematology/Oncology, Department of Pediatrics, The Hospital for Sick Children, University of Toronto, Toronto, Ontario, Canada; Division of Hematology/Oncology, Department of Pediatrics, The Hospital for Sick Children, University of Toronto, Toronto, Ontario, Canada; Division of Hematology/Oncology, Department of Pediatrics, The Hospital for Sick Children, University of Toronto, Toronto, Ontario, Canada; Division of Neurosurgery, School of Medicine, Johns Hopkins University, Baltimore, Maryland, USA; Division of Pathology, School of Medicine, Johns Hopkins University, Baltimore, Maryland, USA; Division of Neuroradiology, The Hospital for Sick Children, University of Toronto, Toronto, Ontario, Canada; Department of Radiation Oncology, Mayo Clinic, Rochester, MN, USA; Department of Pediatrics, Columbia University Irving Medical Center, New York, New York, USA; Division of Hematology/Oncology, Department of Pediatrics, The Hospital for Sick Children, University of Toronto, Toronto, Ontario, Canada; The Brain Tumor Institute, Center for Neuroscience and Behavioral Medicine, Children’s National Health System, Washington, DC, USA

**Keywords:** intrathecal topotecan, metronomic therapy, pineoblastoma, RB1

Pineoblastomas are rare CNS embryonal tumors, accounting for 30% of pineal tumors, and often affecting infants and young children.^1–3^ Historically, tumors were grouped with supratentorial primitive neuroectodermal tumors and treated on high-risk brain tumor protocols with intensive multi-modality treatments, often including craniospinal irradiation (CSI).^2^ Four distinct molecular subgroups of pineoblastoma have recently been identified (PB-miRNA1, PB-miRNA2, PB-MYC/FOXR2, and PB-RB1).^1,3^ PB-miRNA1 and PB-miRNA2 affect older children (median 8.5-11.8 years) while PB-RB1 and PB-MYC/FOXR2 affect younger children (median 1.4-2.1 years). Patients <3 years old have a 5-year overall survival (OS) of 24.2% compared with 77.0% in older children.^1,2^ We present a diagnostically challenging case of an infant with a suprasellar tumor found to be a pineoblastoma, PB-RB1 subgroup. This case illustrates challenges in treating PB-RB1, which harbor a poor prognosis and limited radiation options due to patients’ young age. We discuss strategies to limit treatment-related toxicities while improving outcomes for young children with PB-RB1.

## Case Report

A 6-month-old former 39-week gestational age male presented with left third and sixth cranial nerve palsies and orbital swelling. Abnormal eye movements were first noted 3 weeks prior to presentation. The initial examination was significant for left lateral eyelid swelling and ptosis with restricted upward and outward gaze on examination and after eliciting a vestibulo-ocular reflex. Family history was positive for the patient’s mother and aunt with post-axial polydactyly, and paternal grandmother with polymyositis and dermatomyositis. No brain tumors or other cancers in the family. The patient had no significant past medical history.

**Figure 1.**
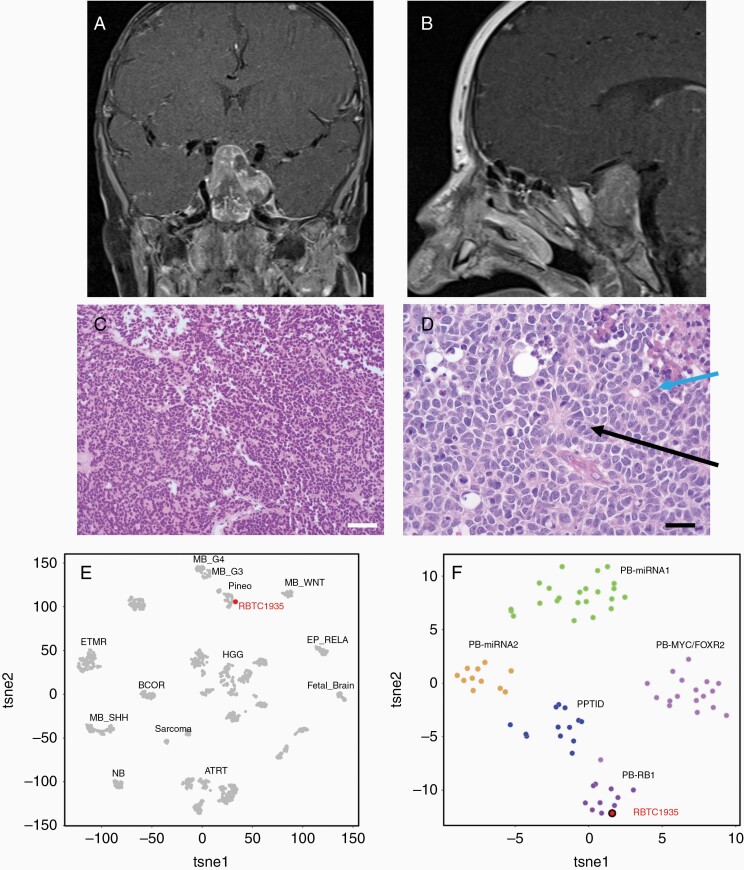
(A) T1-weighted coronal post-contrast MRI at time of diagnosis demonstrates a lobulated T1 isointense heterogeneously enhancing sellar and suprasellar mass measuring 3.0 × 2.0 × 4.1 cm. (B) T1-weighted sagittal post-contrast images further define the lesion. (C) H&E staining of the initial pathology identifies a hypercellular neoplasm composed of small round blue cells with brisk mitotic activity, necrosis, and apoptosis consistent with WHO grade IV CNS embryonal tumor. White scale bar represents 250 µm. (D) Homer Wright rosettes (black arrow) and Flexner-Wintersteiner rosettes (blue arrow) were identified and are consistent with retinoblastic differentiation. Black scale bar represents 100 µm. (E) *t-*distributed stochastic neighbor embedding (*t*SNE) plots of DNA methylation clustering patterns. The patient’s tumor (labeled RBTC1935 in red) clustered with pineoblastoma tumors. (F) Further analysis demonstrated that the tumor clustered with the RB subgroup of pineoblastoma.

MRI revealed a lobulated T1 isointense heterogeneously enhancing sellar and suprasellar mass measuring 3.0 × 2.0 × 4.1 cm^3^ (Figure 1A and B). No metastatic deposits were identified. The patient underwent a partial resection of the tumor with a right modified orbitozygomatic craniotomy and AxiEM-frameless stereotactic image guidance. Complete resection was not possible due to concerns for the patient’s safety debulking a tumor in the suprasellar location. Histology revealed a hypercellular neoplasm composed of small round blue cells with brisk mitotic activity, necrosis, and apoptosis (Figure 1C). There were Homer Wright rosettes and Flexner-Wintersteiner rosettes (Figure 1D). Tumor cells showed strong synaptophysin expression. INI1 was retained, and NKX2.2 and CD99 were negative. Differential diagnosis at this time consisted of a CNS embryonal tumor NOS, pineoblastoma, and a *SMARCA4*-mutated AT/RT. Comprehensive genomic tumor profiling identified mutations in *RB1* p.R255 and *MUTYH* p.G382D. DNA methylation-based tumor profiling was performed at the National Cancer Institute and the tumor clustered with pineoblastoma group A/intracranial retinoblastoma. Further methylation cluster analysis was performed at the Hospital for Sick Children, Toronto, against their internal reference dataset. This analysis classified the tumor in the RB subgroup of pineoblastoma (Figure 1E and F).^1^ Final staging CSF was negative.

The patient started treatment with intensive chemotherapy with the intention to treat with 3 cycles of induction chemotherapy followed by tandem autologous bone marrow transplants (Table 1). This treatment is designed to delay the need for radiation in infants with high-risk brain tumors. The patient’s therapy was complicated by life-threatening toxicities including pericardial effusion, pulseless electrical activity/cardiac arrest, and thrombotic microangiopathy. The patient recovered from these toxicities and MRI after 2 cycles of induction chemotherapy demonstrated a complete remission (CR).

**Table 1.** Summary of Patient’s Treatment Course With Toxicities and Complications

Line of Therapy	Type of Therapy	Chemotherapy	Complications	Reason to Change Therapy
1	HEADSTART IV	• Vincristine • Cisplatin • Etoposide • Cyclophosphamide • Methotrexate	• Bronchospasms • Pericardial effusion • Pulseless electrical activity (PEA) bradycardic arrest	Toxicity
2	ACNS0334 Reg. A	• Vincristine • Cisplatin • Etopophos • 50% Cyclophosphamide	• Thrombotic microangiopathy	Toxicity
3	Metronomic	Alternating oral: • Cyclophosphamide • Etoposide • Temozolomide Combined with alternating: • Celecoxib • Isotretinoin Combined with monthly: • IT Topotecan	• None	Progressive disease
4	Salvage chemotherapy	• Ifosfamide • Carboplatin • Etopophos	• Partial seizures	Toxicity
5	Salvage chemotherapy	• Cyclophosphamide • Carboplatin • Etopophos	• None	Progressive disease
6	Targeted therapy	• Gefitinib	• None	Progressive disease

Abbreviation: IT, intrathecal.

Given these severe toxicities, the patient was transitioned to metronomic therapy with the goal of maintaining remission until the patient could better tolerate the re-intensification of therapy. His regimen consisted of 21-day cycles alternating oral temozolomide, cyclophosphamide, and etoposide in combination with alternating cycles of celecoxib and isotretinoin. Intrathecal (IT) topotecan was administered every 4 weeks. Surveillance imaging demonstrated CR throughout therapy. He tolerated 9 cycles of therapy with only minor toxicities including occasional neutropenia and intermittent nausea. We were next planning to stop metronomic therapy and trial re-intensification of therapy to help maintain the patient’s complete response.

**Figure 2.**
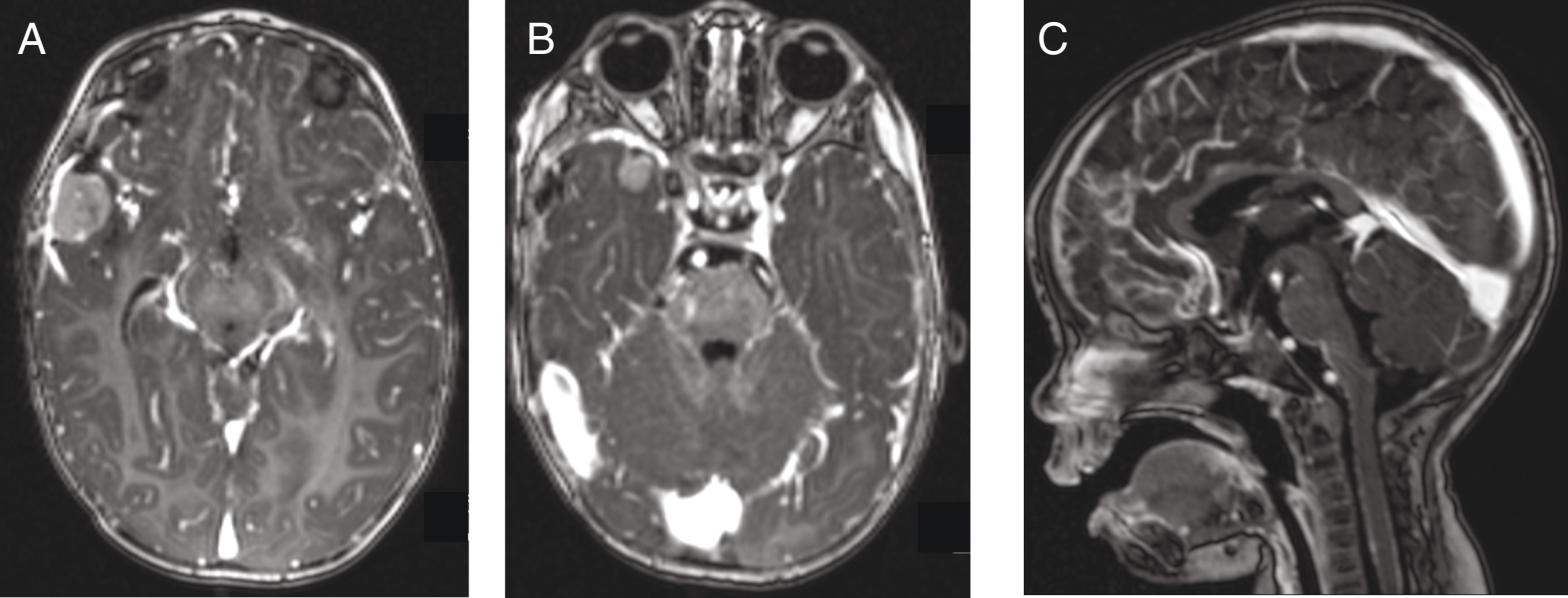
(A) T1-weighted axial post-contrast MRI at the time of first relapse demonstrating 2.2 × 1.8 cm right parietal lobe mass. (B) T1-weighted axial post-contrast MRI demonstrating 1.1 × 1.1 cm right temporal polar area mass. (C) T1-weighted sagittal post-contrast MRI demonstrating leptomeningeal thickening and enhancement most notable along the midbrain and brainstem.

The MRI at the end of metronomic therapy revealed tumor recurrence with diffuse CNS metastases and leptomeningeal disease (Figure 2). Salvage chemotherapy was initiated with ifosfamide, carboplatin, and etoposide (ICE). The course was complicated by partial seizures related to ifosfamide neurotoxicity. The patient then transitioned to cyclophosphamide, carboplatin, and etoposide (CCE) and received 2 additional cycles without significant toxicities. After these 2 cycles of CCE, MRI demonstrated a second CR.

The patient was administered a third cycle of CCE with stem cell collection to support the further intensification of chemotherapy with stem cell rescue. However, mobilization due to heavy pre-treatment did not produce sufficient cells to support an autologous bone marrow transplant. While recovering from this cycle, the patient developed vomiting, irritability, and ataxia. MRI revealed the diffuse leptomeningeal disease. The patient subsequently started Gefitinib for disease-directed therapy but his cancer rapidly progressed, and the patient died from progressive disease 1 week after starting Gefitinib and about 18 months after diagnosis.

## Discussion

The advent of new molecular data has facilitated more precise classification of pediatric brain tumors and has helped define distinct subgroups of tumors that respond poorly to standard therapies. These molecular techniques can be especially helpful in CNS embryonal tumors, which can be located in many different areas of the brain, and have few distinct histological features. Radiology and histologic evaluation of our patient’s tumor demonstrated an embryonal tumor in the suprasellar location. PB-RB1 rarely presents in this location and have few histological features distinguishing them from other CNS embryonal tumors. Comprehensive genomic profiling and DNA methylation-based tumor profiling were therefore critical for making the diagnosis in this case and were important in guiding subsequent treatment decisions.

Treatment for infants with PB-RB1 poses several challenges illustrated in this case report. Intensive multi-modality therapy is considered a standard of care for pineoblastoma. However, PB-RB1 primarily affects infants where chemotherapy-related toxicities are more variable and often more severe than in older patients. Our patient suffered numerous toxicities from these standard therapies leading to frequent treatment delays and dose reductions, which can also limit their efficacy. This case highlights the importance of developing alternative treatment strategies for infants who cannot tolerate standard therapies. In this case, we considered targeted radioimmunotherapy with ^131^I-Omburtamab. This treatment can be administered via the intraventricular (IVT) route using an Ommaya reservoir or a programmable ventriculoperitoneal shunt and has been shown to be safe and feasible. However, our patient’s disease relapsed before we could consolidate with ^131^I-Omburtamab.

CSI is an additional therapeutic option that can be highly effective for pineoblastoma including PB-RB1. Patients receiving upfront radiotherapy have a 5-year event-free survival (EFS) of 58.8% and OS of 71% compared to 10% and 40% for patients who do not receive RT.^1^ However, RT is often avoided in the treatment of infants like our patient, due to its devastating impact on neurocognitive outcomes. Developing a safe mechanism to incorporate RT into infant treatment plans could help improve outcomes. Less toxic RT options like highly conformal RT techniques^4^ or proton therapy^5^ may have a more limited impact on disease outcomes due to frequent distant recurrences in RB-PB1.^2^ Alternatives for young children include combining upfront conformal RT with treatments that can limit distant spread and recurrence of disease or including additional therapies to delay the need for CSI until patients are older and can better tolerate its late effects.

IT or IVT chemotherapy is often incorporated into pediatric brain tumor treatment regimens as a mechanism to bypass the blood-brain barrier.^6^ This treatment strategy helps treat or prevent leptomeningeal disease but concentrations of drug decline significantly millimeters into white matter, limiting its efficacy to treat intraparenchymal tumors.^7^ Topotecan is an inhibitor of topoisomerase I and has been used for patients with retinoblastoma and recurrent CNS embryonal tumors with some considerable treatment responses.^8^ Topotecan is tolerated well in pediatric patients when administered IT or IVT.^9^ Given this efficacy and safety data in pediatrics, we treated our patient with IT topotecan. Therapy was well tolerated and helped prevent recurrent disease for more than 6 months. Topotecan administered through an Ommaya catheter may be more effective than IT therapy in treating brain tumors. The Ommaya catheter facilitates drug administration directly into the ventricular reservoir and allows for more reliable drug delivery, homogeneous drug distribution, and higher drug concentration in the subarachnoid space compared with lumbar punctures.^7^ Use of an Ommaya catheter does not require sedation which can facilitate more frequent dosing of the chemotherapy. Including IVT topotecan in upfront treatment regimens may prevent leptomeningeal tumor dissemination and could improve the efficacy of focal proton RT in PB-RB1. Alternatively, IVT topotecan could be used as part of a maintenance treatment regimen.

Maintenance chemotherapy might help delay the need for CSI in young patients with pineoblastoma. Metronomic therapy is one strategy utilized as a maintenance regimen in similar patient populations. This treatment primarily affects the tumor microenvironment by inhibiting angiogenesis and stimulating an immune response rather than by direct antitumor effects.^10^ These regimens include low-dose oral chemotherapies, such as etoposide, cyclophosphamide, and temozolomide in combination with celecoxib and isotretinoin or thalidomide. This treatment is well tolerated in a heavily pre-treated population and can delay tumor recurrences.^10^ Our patient tolerated the combination of metronomic therapy with IT topotecan without any severe toxicities which has been tested in young patients with CNS embryonal tumors, was tolerated with minimal toxicities, and provided some therapeutic benefits.^11^

Targeted molecular therapies might also help treat these aggressive infantile tumors and reduce treatment-related toxicities. High throughput genetic and epigenetic studies have identified increased expression in epidermal growth factor receptors (EGFR) in the RB subgroup of pineoblastoma. Endersby et al demonstrated the erythroblastic leukemia viral oncogene homolog inhibitor (pan-ERBB inhibitor) Dacomitinib inhibited EGFR signaling in in vitro models of pineoblastoma and had efficacy in orthotopic models of pediatric brain tumors.^12^ Many EGFR inhibitors have been tested in children. We treated our patient with Gefitinib given the extensive safety data in pediatric patients and efficacy in treating pediatric brain tumors. Newer agents like Dacomitinib or lapatinib may more fully inhibit EGFR signaling but require further clinical trials to test for safety in pediatric patients.

In silico drug screening in a recently established Rb-deficient murine pineoblastoma model identified nortriptyline as a potential therapy for this subgroup of pineoblastoma. Rb-deficient tumors were especially sensitive to nortriptyline-induced lysosome disruption and autophagy-induced cell death.^13^ While direct targeting of loss of function mutations is not possible, the deletion may lead to unique dependencies or vulnerabilities that can be targeted. In the case of RB-mutated tumors, rapid cell cycle progression leads to replication stress and mitotic checkpoint abnormalities that increase the activity of aurora kinases and drive a dependency on polo-like kinase 1 (PLK1) and checkpoint kinase 1 (CHK1).^14,15^ Inhibitors of these factors have demonstrated efficacy against orthotopic xenograft models of RB-mutated tumors. Alisertib, Volasertib, and Prexasertib inhibit aurora kinase, PLK1, and CHK1, respectively, and have been tested in pediatric clinical trials. These agents can be used within the backbone of standard therapy to improve outcomes for the RB subgroup of pineoblastoma or as part of a maintenance regimen to delay or prevent the need for CSI.

## Conclusion

We present the case of an infant with a suprasellar pineoblastoma with subgroup PB-RB1. Comprehensive genomic tumor profiling and DNA methylation-based tumor profiling were critical to classifying the tumor as PB-RB1 molecular subgroup. CSI was considered to be too harmful for this young patient and standard therapies led to severe, life-threatening toxicities which were insufficient to induce a long-term CR. This prompts the need for improved treatment strategies in this population of patients with PB-RB1. Strategies to improve therapy include combining highly conformal proton therapy with IVT topotecan or other therapies that prevent distant sites of recurrences or designing a maintenance therapy regimen with metronomic therapy or immunotherapy. More precise disease-directed therapies targeting RB-induced vulnerabilities, such as the EGFR pathway, aurora kinases, PLK1, or CHK1 could be investigated as additions to the backbone of standard therapies or as a maintenance regimen to improve outcomes.
